# The Story of Managed Ventricular Pacing

**DOI:** 10.19102/icrm.2021.120804

**Published:** 2021-08-15

**Authors:** David A. Casavant, Paul Belk

**Affiliations:** ^1^Unaffiliated; ^2^Boston Scientific, Natick, MA, USA

**Keywords:** Cardiac pacing, Managed Ventricular Pacing, mode switching

## Abstract

A significant milestone in cardiac pacing occurred approximately two decades ago, when the primary operating mode was reimagined to more closely mimic normal top-down cardiac activation. When introduced, Managed Ventricular Pacing (MVP™; Medtronic, Minneapolis, MN, USA) was an unprecedented dual-chamber mode as it preferentially paced the right atrium in the AAI/R mode and simultaneously protected against transient heart block, albeit only in the instance of dropped ventricular beats. At the time, dual-chamber DDD/R with atrial-based timing and programmable atrioventricular delay was state of the art. MVP™ “unlocked” conventional dual-chamber pacing by not consistently requiring a 1:1 atrioventricular relationship during its primary operating mode (ie, AAI/R+). Ultimately, MVP™ emerged as a primitive means to promote His–Purkinje activation, and it is not a coincidence that its roots can be traced back to first-in-man permanent His-bundle pacing.

## Introduction

*“Don’t be trapped by dogma—which is living with the results of other people’s thinking. Don’t let the noise of others’ opinions drown out your inner voice. And, most importantly, have the courage to follow your heart and intuition.”—Steve Jobs*

Managed Ventricular Pacing (MVP™; Medtronic, Inc., Minneapolis, MN, USA) was conceived as a permanent pacing modality in 1999, nearly three decades after an atrial lead was added to permanent pacing and dual-chamber pacemakers were commercialized.^1^ At the time, pacemakers were indicated in more than two-thirds of paced patients to correct symptoms resulting from abnormally slow origination of impulses in the sinoatrial node. Yet, as pacemakers were developed from the bottom up, starting with a VOO mode, they incrementally evolved to pace in a dual-chamber fashion such that every cardiac cycle ended with a ventricular sensed or paced event. Atrioventricular (AV) hysteresis, introduced in 1995, periodically extended AV delay for one beat and represented the first attempt to encourage intrinsic conduction. More sophisticated AV search hysteresis algorithms became available in 1999 and strived to discover intrinsic conduction over a series of beats. However, as aggressive as these algorithms were, they were limited by the programming confines of DDD/R pacing, including, most notably, the restrictions imposed to avoid 2:1 pacemaker block. MVP™ was revolutionary as it differed from all other dual-chamber modes and relaxed the requirement that all atrial events be followed by a ventricular event. In doing so, ventricular pacing was practically eliminated in all patients except those with persistent complete heart block.^2^

The approval of MVP™ by the United States Food and Drug Administration in August 2004 did not require a large, prospective randomized trial. Instead, it was approved following two relatively small temporary software download trials involving a total of 211 patients (30 patients using the Gem III DR system and 181 using the Marquis DR system, respectively; both Medtronic). Both trials proved MVP™ to be safe and highly effective in reducing right ventricular (RV) pacing, with no clinical endpoints. The commercial release was bolstered by emerging and compelling evidence showing the harmful effects of RV overpacing. The most compelling trial findings came from the Dual Chamber and VVI Implantable Defibrillator (DAVID) trial^3^ and the Mode Selection Trial (MOST) and its substudy^4^ published between 2002 and 2003. In combination, these trials provided incontrovertible evidence that ventricular overpacing contributed to heart failure (HF) and atrial fibrillation (AF). Another important contemporaneous realization came from evidence that iatrogenic left bundle branch block created by RV pacing activation was equally harmful in patients with congestive HF without left bundle branch block.^5^ As such, results from early cardiac resynchronization therapy trials further contributed to the early adoption of MVP™.^6,7^

## The MVP™ story

**Figure 1:**
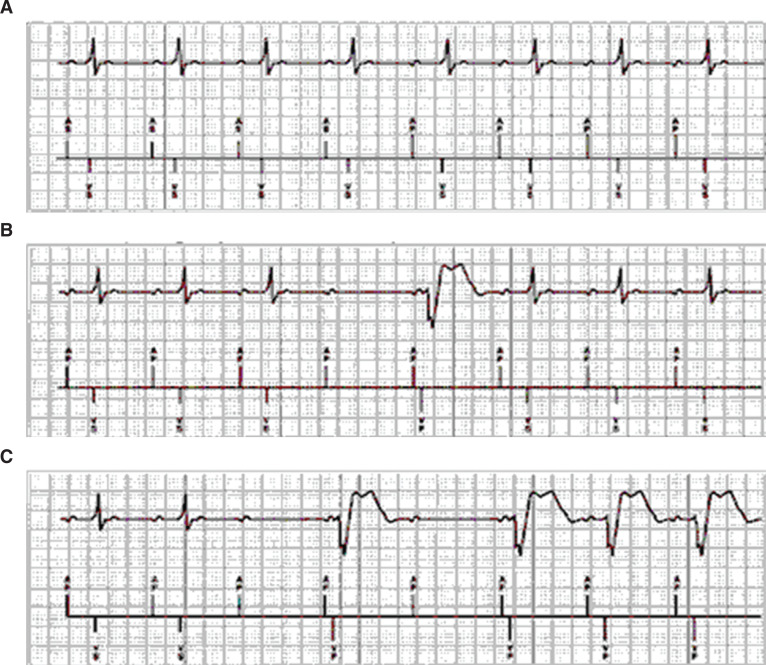
**A:** ECG and Marker Channel™ captured in the AAI/R+ mode, MVP™’s predominant operating mode in patients with intact conduction. **B:** Example showing issuance of a backup ventricular pace following a singular nonconducted atrial event. **C**: Conversion to a temporary DDD/R mode in the event of high-degree heart block (ie, two of four intervals without a ventricular sense). Not shown is the AAI/R+ restoration method, which involves dropping a ventricular pace periodically and which is deemed successful if a ventricular sense occurs prior to the next atrial event.

**Figure 2:**
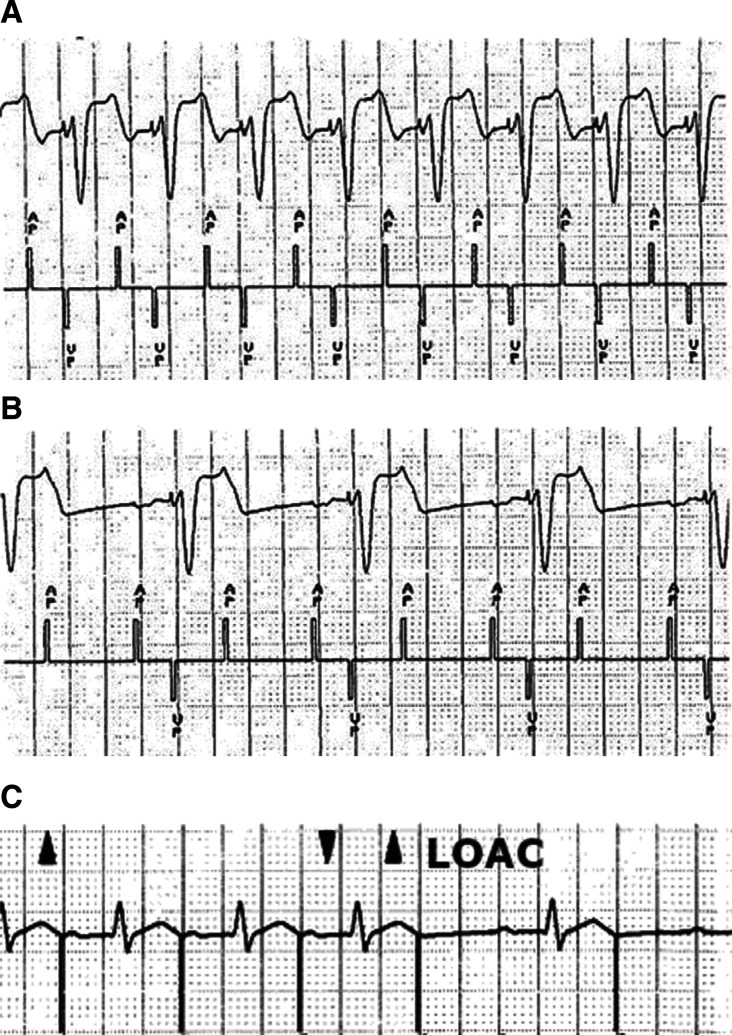
**A:** ECG/marker strip taken during 1:1 AV pacing at a rate of 120 ppm. Atrial capture verification was complicated by an overlap of atrial pacing and the terminal end of the ventricular paced complex. **B:** ECG/marker strip taken during 2:1 AV pacing at a rate of 120 ppm. Note that, by liberating isoelectric time, atrial capture assessment on alternating intervals is visually unhampered. **C:** Sustained ADI pacing in the event of 1:1 AV conduction. Loss of atrial capture (LOAC) is indicated by an abrupt and sustained fallback in heart rate with the appearance of native p-waves on the ECG.

The true origin of MVP™ can be traced back to 1995, when its predominant AAI+ operation was first described by David Casavant, a Boston-based field clinical engineer, as a means to safely facilitate atrial capture threshold determination **(Figure 1)**.^8^ Prior to pacemaker systems having a real-time, multichannel electrocardiogram (ECG) and event marker display, the assessment of atrial capture during attended follow-up was difficult and virtually impossible in patients with sinus tachycardia or frequent premature atrial contractions and complete heart block **(Figure 2A)**. An innovation known as “facilitated atrial pacing threshold testing” (FAPTT) emerged as a means to more easily perform the atrial threshold test by way of 2:1 AV pacing. Doing so thereby extends the isoelectric time and allowed visualization of atrial capture on alternating beats **(Figure 2B)**.^8^ Notably, the invention maintained atrial-only pacing in the event of intrinsic conduction and thereby allowed for atrial capture inference during sustained overdrive atrial pacing **(Figure 2C)**.

The timeline and motivations for the development of MVP™, highlighting the controversies and uncertainties that surrounded the conception of a feature that continues to be considered standard of care, are described henceforth.

### Conceptualization

In the case of MVP™, a common falsehood is that the development of MVP™ was a response to multicenter trials—most notably, the DAVID trial—which were designed to confirm the general understanding that excess ventricular pacing was harmful. When MVP™ was initially developed in 1999 and 2000, the idea of harm from ventricular pacing was a very small minority opinion. In fact, the original purpose of the DAVID trial was to establish the superiority of dual-chamber pacing, and the results of the DAVID trial (which was terminated early) represented only the beginning of a reevaluation of that idea.

MVP™ has been recognized as a disruptive innovation that was introduced well before its time and continues to be prescribed as a mainline treatment for patients with sinus node dysfunction and/or paroxysmal heart block. At the time of its conception in 1999, the most significant clinical evidence that AAI was superior to DDD was from a small (ie, 177 patients), randomized, prospective (DANPACE I) trial performed in Denmark, which showed a significantly lower incidence of AF in patients paced with the AAI mode.^9^ Although AAI-mode pacing was prescribed routinely in some European countries, atrial-only pacing never obtained a foothold in the United States, with few American device implanters willing to risk the possibility of frank syncope in a pacemaker patient. At the time that evidence of AAI pacing’s superiority in sick sinus syndrome patients began to mount, insertion of an RV pacing lead had already become an established practice. In a country having a robust health care system and a degree of litigiousness, single-lead ventricular pacing simply evolved directly to dual-lead AV pacing systems and, in the United States, little consideration was given to atrial-only pacing. In fact, AAI pacing was declared extinct in 2001.^10^ The best option for sick sinus syndrome patients at the time was programming DDD/R or DDI/R with a long AV (eg, 350 ms) delay, but static, long AV delays introduced the potential for retrograde conduction and resultant arrhythmias, including pacemaker-mediated tachycardia and repetitive non-reentrant ventriculoatrial synchrony.

MVP™ was fully described before the plan for the DAVID trial was publicly announced^11^ and before the MOST substudy results were published.^4^ Interestingly, the MOST and DAVID trials hypothesized that dual-chamber DDD pacing, then considered the universal pacing mode, was likely to prove superior. Ultimately, the MOST trial showed that DDD pacing was equivocal to VVIR with respect to the combined endpoint of death or nonfatal stroke and confirmed that the degree of ventricular pacing in both the DDD and VVIR modes contributed to increased HF hospitalizations and AF, while the DAVID trial showed that DDD pacing increased the combined endpoint of death or HF hospitalization relative to backup VVVI pacing in patients with implantable cardioverter-defibrillators (ICDs) having compromised left ventricular function. It was ultimately the combined results from DANPACE I, the MOST substudy, and DAVID, together with the coincidental realization of the harmful effects of dyssynchronous ventricular activation gained from early cardiac resynchronization therapy trials,^6,7,12–16^ that cinched the destiny of MVP™.

Although dual-chamber pacing had become widely accepted as the “physiologic” or “universal” pacing modality in the mid- to late 1990s, the existence of excessive ventricular pacing was troubling to Casavant and Paul Belk, a Medtronic field scientist stationed at Boston’s Beth Israel Hospital. Casavant had worked extensively with Dr. Pramod Deshmukh, widely recognized as the electrophysiologist who pioneered permanent His-bundle pacing. In the early 1990s, Dr. Deshmukh had realized that pacemaker implantation in patients with compromised left ventricular function often seemed to exacerbate their HF and accelerate mortality. More importantly, though, he understood why. In 1968, researchers had shown that those ventricles that were activated normally via temporary His bundle pacing functioned better.^17^ As the collaborator and coauthor of the landmark publication describing first-in-man permanent His-bundle pacing, Casavant learned the paramount importance of preserving normal activation in pacemaker-indicated patients with failing hearts.^18^ Published studies suggesting adverse consequences from the use of conventional DDD/R pacemakers already existed but were largely ignored.^19–23^ Most notably, a publication in 1993 from a large single-center study involving 557 patients hospitalized for HF showed that the risk of non–sudden cardiac death at one year was 48% higher in patients with pacemakers.^24^ Belk had done his PhD work in developing a finite element model of ventricular arrhythmias and had concluded that abnormal ventricular activation increased ventricular tachyarrhythmia susceptibility in ICD-indicated patients. Under the guidance of Dr. Mark E. Josephson, he designed a simple protocol in patients being evaluated for ICDs and showed that ventricular tachycardia (VT) was more readily induced when premature ventricular stimuli (ie, S2, S3, S4) were delivered in the wake of a ventricular paced (S1) drive train than when premature ventricular stimuli were delivered at the same coupling intervals following a narrow QRS drive train produced by atrial pacing.^25^

MVP™ was conceived in a clinical environment in Boston without substantial input from the vast array of Medtronic in-house engineers, scientists, marketing, and product planners. The widespread realization that ventricular pacing was possibly not benign began following a presentation of the MOST results as a late-breaking clinical trial at the North American Pacing and Electrophysiology Society Meeting in 2001. Thereafter, it became a higher priority for pacemaker practitioners to avoid ventricular pacing. Programming long AV delays within the constraints of DDD/R pacing, mainly to avoid pacemaker 2:1 block at elevated sinus rates, however, was often a difficult task. Although pacemakers with AV search hysteresis DDD/R-mode algorithms did prove to be quite effective in reducing ventricular pacing in pacemaker recipients,^26–28^ they had not yet been implemented or even tested on a dual-chamber ICD platform. In fact, initial DDD ICDs introduced in the United States in 1998 (Ventak AV™; Guidant, Indianapolis, IN, USA), 1999 (Gem DR™; Medtronic), and 2000 (Photon DR; St. Jude Medical, St. Paul, MN, USA), respectively, did not incorporate AV search algorithms.

**Figure 3:**
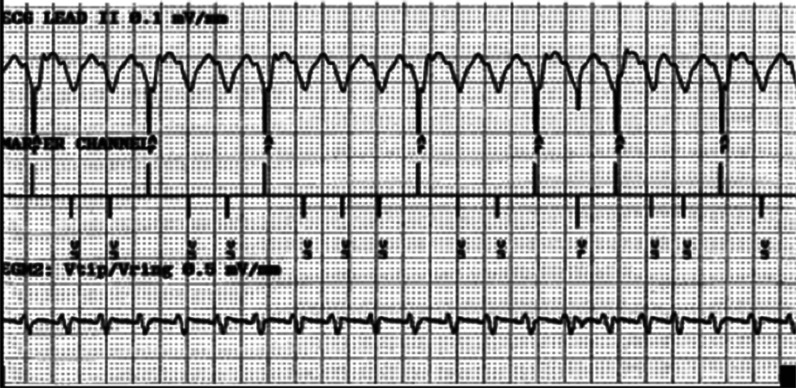
Simulated failure to detect rapid VT due to long AV delay programming and interference from post-Ap and post-Vp ventricular blanking periods during sensor-driven pacing at 100 bpm and during VT at a rate of 200 bpm. Programmed DDDR, lower rate (LR) =60 bpm, upper sensor rate (USR) = 130 bpm, paced AV (PAV) = 280 ms, tachycardia detection interval (TDI) = 280 ms, and fibrillation detection interval (FDI) = 320 ms (VS markers are not denoted as TS as the above could only be simulated). UR: upper rate.

The most important influence on the “AV delay-less” design of MVP™ was that it provided a means to overcome a mandate imposed by Medtronic’s dual-chamber ICD designers that the VP–AP interval “fit” within the VT detection zone at all times so as to not interfere with VT detection due to same-chamber and cross-chamber post-pace blanking periods. Such a scenario could easily be demonstrated on a simulator **(Figure 3)**. At the time, most electrophysiologists and industry experts maintained that the primary function of ICDs was to protect patients from malignant ventricular tachyarrhythmias and accepted that some degree of ventricular overpacing was acceptable.

### Development efforts

To fully define MVP™ as a permanent pacing modality, the following enhancements were added to the FAPTT algorithm: (1) an AV delay of 80 ms following a nonconducted atrial event, which was chosen for being the shortest AV delay known to be asymptomatic during the clinical evaluation of Medtronic’s noncompetitive atrial pacing feature in the Thera™ DR pacemaker (Medtronic); (2) a postatrial refractory period of 600 ms for rates below 75 bpm and 75% of the ventricular interval for rates above 75 bpm, both of which were somewhat arbitrarily chosen to delineate premature atrial contractions from “physiologic” atrial sensed events originating from the sinus node; (3) a reverse mode switching AA/IR+ to DDD/R in the event of 2:1 block or rhythms having a ratio of A:V events below 4:3; (4) a mode switching to DDI/R from either AAI/R+ or DDD/R, in the event of AF; (5) periodic attempts to restore AAI/R+ operation following DDD/R reversion by withholding singular ventricular paced events at geometrically increasing intervals (eg, one minute, two minutes, four minutes, eight minutes—up to 16 hours); and (6) a premature ventricular contraction (PVC) response during AAI/R+ operation that suspends atrial pacing to eliminate potential VT detection interference from postatrial and ventricular cross-blanking following PVCs and PVC runs. **Figure 1** demonstrates basic MVP™ operation.

**Figure 4:**
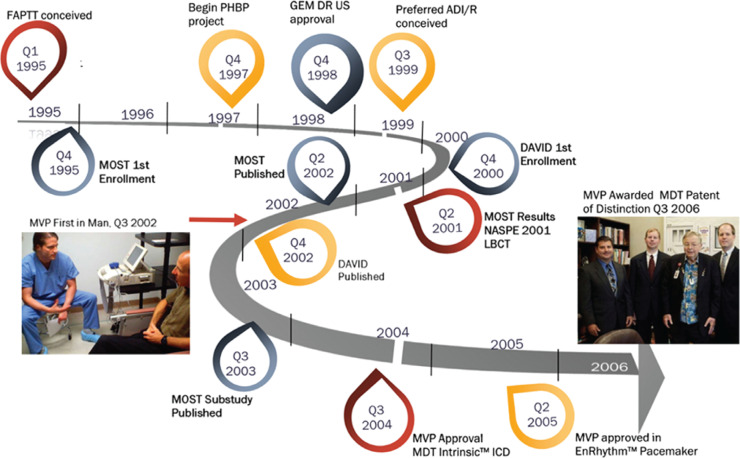
Timeline showing the evolution of MVP™ from preconception to final release. FAPTT refers to a facilitated atrial threshold test. Preferred ADI/R was the original name for MVP™. On the left, Dr. Michael O. Sweeney is pictured with the first MVP™ patient during the Gem III DR download study. On the right, David Casavant, Paul Belk, Medtronic founder Earl Bakken, and Tom Mullen are pictured after receiving the Medtronic Patent of Distinction Award, the 12^th^ award granted in Medtronic’s history of more than 65 years recognizing disruptive innovation.

In the end, four engineers can be credited as the inventors of MVP™.^29^ Casavant and Belk were the primary architects, while Tom Mullen, PhD, contributed solutions to several of the pace timing issues necessary to make the final MVP™ design reliable in the general pacing population and was also instrumental in the design, data analysis, and publication of the MVP Gem III DR download study.^30^ The fourth inventor, John Stroebel, a veteran Medtronic system engineer, contributed to the final design of MVP™ and conceived a sophisticated “under-the-hood” method to implement MVP™ as a download algorithm in the Gem III ICD. His contribution expedited the clinical evaluation of the algorithm and hastened commercial approval for MVP™. The timeline for MVP™ conception to market approval is depicted in **Figure 4**.

Of course, the complete list of MVP™ contributors is vast and includes all those who participated in the planning, design, development, and regulatory approval. MVP™ received regulatory approval based on one-week data obtained from 30 patients of Dr. Michael O. Sweeney, an electrophysiologist from Boston’s Brigham and Women’s Hospital (BWH), and on multicenter data from 206 patients having the MVP™ algorithm temporarily downloaded as “RAMware” into the Marquis DR ICD.^31^ The commercial release occurred in 2004 with the launch of the Medtronic Intrinsic™ ICD.^32^ The MVP™ story would not be complete without also crediting Dr. Sweeney for coincidentally coming to the nearly simultaneous realization that RV overpacing was harmful. Dr. Sweeney’s first recognition of the deleterious impact of RV pacing came during analyses of the results from the landmark MOST study, which failed to show a significant benefit of DDD over VVIR pacing,^33^ and his extensive subanalyses showed that ventricular overpacing, either in the DDD or VVIR mode, contributed to incidence rates of HF hospitalization and AF in pacemaker patients.^4^ After joining Medtronic as a consultant on the MVP™ technology, his relentless advocacy and teachings solidified his position as a primary advisor and investigator of MVP™. Dr. Sweeney oversaw the first-in-man applications of MVP™ at BWH in late 2002 and his established authority as a key opinion leader greatly influenced expedited testing and commercial approval of MVP™. Dr. Sweeney is primarily responsible for ultimately convincing the worldwide pacing community that MVP™ was a solution to DDD/R pacing and that the maintenance of cardiac harmonious activation via its innate “infinite virtual electrode” is highly preferable over optimal AV timing.

## Other AAI↔DDD algorithms

Near the time of MVP™’s approval or soon thereafter, all other cardiac rhythm device companies secured approval for commercial release of more assertive ventricular pace minimization algorithms. AAISafeR^®^ and AAISafeR 2 (ELA/Sorin, Milan, Italy), released internationally in 2003 and 2005, respectively, were the only other pacing modes having AAI/R↔DDD/R functionality similar to MVP™ at the time. (MVP™ and AAISafeR 2 DDD/AAI modes were conceived by autonomous inventors from independent companies working in the United States and France, respectively.) Strategies used by other manufacturers included Auto-intrinsic Conduction Search^®^ (AICS) in 2006 (St. Jude Medical), Ventricular Intrinsic Preference^®^ (VIP) in 2007 (St. Jude Medical), AV Search Hysteresis^®^ (AVSH) in 2007 (Guidant), Vp Suppression^®^ and Intrinsic Rhythm Search^®^ (IRSplus) in 2010 (Biotronik, Berlin, Germany), and Rhythm IQ^®^ in 2012 (Boston Scientific, Natick, MA, USA).

### MVP™ 2.0

The second generation of MVP™ was implemented in cardiac rhythm devices released beginning in 2017 with slight modifications, including an optional programmable limit for the longest allowed A–R interval and an enhancement to lessen the duration of long V–V intervals and the severity of short–long–short (S–L–S). This second version of MVP™ employs a dynamic, adjusting A–A interval and a programmable maximum AV interval limit such that switching to DDD/R occurs if two of four Ap–Vs or As–Vs intervals exceed this limit.^34,35^

## Final perspectives

Many agree that the rapid acceptance of MVP™ was largely due to its simplicity in that it more closely mimicked normal activation of the heart and allowed for the management of non–pacemaker-indicated rhythms—including first-degree and second-degree Mobitz I, type I “Wenckebach” heart block. This simplicity was enhanced by its lack of programmable parameters. MVP™ was introduced as an ON/OFF feature, a characteristic the inventors vehemently defended, thereby avoiding the “feature creep” that often results from the desire to appease all. The naming of this mode is owed to Dr. Sweeney.

In some ways, MVP™ can be viewed as a precursor to future permanent His-bundle pacemaker modalities (eg, sequential A–H pacing systems) given that both favor normal ventricular activation. In fact, it is being employed on occasion for patients with intermittent heart block receiving A–H pacing systems by programming short AV delays consistent with normal A–H intervals.

MVP™ has seen very high levels of clinical success in prospective, randomized clinical trials. The MVP™ versus VVI 40 pacing trial, performed in an ICD population, similar to the DAVID trial, was stopped for futility as the combined endpoint of worsening HF or all-cause death was equally low in both arms.^31^ The Search AV Extension and Managed Ventricular Pacing for Promoting Atrioventricular Conduction (SAVe PACe) pacemaker trial showed that, in patients paced using a minimal ventricular pacing strategy, the risk of progression to persistent AF was reduced by 40%.^36^ An ensuing multicenter 1,166-patient pacemaker trial known as Minimize Right Ventricular Pacing to Prevent AF and HF (MINERVA) demonstrated that the combined efficacy of MVP™ when prescribed alone—and particularly when combined with atrial antitachycardia pacing—achieved a significant overall reduction in the progression to permanent or persistent AF as compared with among DDDR-paced patients.^37^

Although the MVP™ mode significantly reduced ventricular pacing in the majority of patients, including those having a heart block indication,^2,38^ some criticisms of the mode for allowing occasional pauses that were excessively long have arisen. Sweeney et al. somewhat debunked the notion that pauses were more significant than in other modalities such as backup VVI pacing and showed that, although (nonpaced) S–L–S sequences were permitted more frequently by MVP™, S–L–S sequences terminating with a paced beat were not.^39^

MVP™ has not experienced unconditional patient acceptance. MVP™ has been implicated in VTs in prone patients.^40–43^ However, some patients have reported vague symptoms from pseudo-pacemaker syndrome due to sustained periods of first-degree block with very long P–R intervals. Some patients have not tolerated MVP™ due to symptoms attributed to extended pauses following nonconducted atrial events. Still, experience to date has revealed that only a small minority of MVP™-paced patients have been reprogrammed to conventional DDD/R pacing. Overall, MVP™ 2.0 has addressed concerns.

Despite its shortcomings, MVP™ has been a huge clinical success that resulted from hard work, research, and serendipity. MVP™ represents a product of collaboration amongst engineers and physicians, an attribute that is shared by so many important innovations in medicine.

### Looking to the future

Importantly, MVP™ is not the optimal solution: clinical equipoise is imposed by the competing goals of optimizing AV synchrony while maintaining normal ventricular activation, particularly in patients having long P–R and narrow QRS intervals. In pacemaker-indicated patients, optimizing the AV interval using DDD pacing is only indicated in the event of symptoms due to severe AV decoupling (ie, pseudo-pacemaker syndrome).^44^ In HF patients, physician sentiment continues to favor AAI/R (ie, MVP™) and other modes that limit RV pacing.^45–47^ Ultimately, permanent His-bundle and direct conduction-system pacing will provide better solutions.
